# Association of Nights and Weekends with Survival of Traumatic Out-of-Hospital Cardiac Arrest following Traffic Collisions: Japanese Registry-Based Study

**DOI:** 10.3390/ijerph182312769

**Published:** 2021-12-03

**Authors:** Tatsuma Fukuda, Naoko Ohashi-Fukuda, Hiroshi Sekiguchi, Ryota Inokuchi, Ichiro Kukita

**Affiliations:** 1 Department of Emergency and Critical Care Medicine, Graduate School of Medicine, University of the Ryukyus, Okinawa 903-0215, Japan; hiroshis@med.u-ryukyu.ac.jp (H.S.); kukita@med.u-ryukyu.ac.jp (I.K.); 2 Department of Emergency and Critical Care Medicine, Toranomon Hospital, Tokyo 105-8470, Japan; 3 Department of Acute Medicine, Graduate School of Medicine, The University of Tokyo, Tokyo 113-0033, Japan; fukudan-eme@h.u-tokyo.ac.jp; 4 Department of Health Services Research, University of Tsukuba, Ibaraki 305-8575, Japan; intensivecareunits@gmail.com

**Keywords:** out-of-hospital cardiac arrest, cardiopulmonary resuscitation, trauma, off-duty hours, health care system, work style reform

## Abstract

Background: The process of care for traumatic out-of-hospital cardiac arrest (OHCA) may be different at night and on the weekend. However, little is known about whether the rate of survival after OHCA is affected by the time of day and day of the week. Methods: This observational study analyzed the Japanese government-led nationwide population-based registry data of OHCA patients. Patients who experienced traumatic OHCA following traffic collisions from 2013 to 2017 were included in the study. A multivariable logistic regression model was used to examine the association of both time of day (day/evening vs. night) and day of the week (weekday vs. weekend) with outcomes after traumatic OHCA. Night was defined as 23:00 p.m. to 6:59 a.m., and weekends were defined as Saturday and Sunday. The primary outcome was one-month survival. Results: A total of 8500 patients (mean [SD] age, 57.7 [22.3] years; 68.6% male) were included. 2267 events (26.7%) occurred at night, and 2482 events (29.2%) occurred on weekends. Overall, 173 patients (2.0%) survived one month after OHCA. After adjusting for potential confounders, one-month survival during the day/evening (148/6233 [2.4%]) was significantly higher than during the night (25/2267 [1.1%]) (adjusted OR, 1.95 [95%CI, 1.24–3.07]), whereas there was no significant difference in one-month survival between weekdays (121/6018 [2.0%]) and weekends (52/2482 [2.1%]) (adjusted OR, 0.97 [95%CI, 0.69–1.38]). Conclusions: One-month survival after traumatic OHCA was significantly lower during the night than during the day/evening, although there was no difference in one-month survival between weekdays and weekends. Further studies are warranted to investigate the underlying mechanisms of decreased survival at night.

## 1. Introduction

Out-of-hospital cardiac arrest (OHCA), a leading cause of mortality, is a major public health issue worldwide [1,2,3,4,5]. In Japan, approximately 130,000 cases of OHCA occur annually, with barely 10% survival [6,7,8]. Traumatic OHCA, accounting for less than 10% of all patients with OHCA, has an extremely low survival rate [9,10,11,12,13].

To improve treatment outcomes, Japan’s health policy has promoted the establishment of more emergency and critical care centers that can provide advanced and highly specialized care for critically injured patients 24 h a day, 365 days a year (Table S1 in the Supplementary Materials) [6,14].

Previous OHCA studies demonstrated worse survival during off-duty hours compared to during on-duty hours [15]. The survival rate for OHCA was lower during nights than during days/evenings [16,17], although there was no difference between weekends and weekdays [17]. Conversely, previous studies of trauma did not necessarily demonstrate worse survival during off-duty hours compared to during on-duty hours [15]. The survival rate for trauma was comparable in both nights and days/evenings, nay, and higher during weekends than weekdays [18]. In trauma care, the immediate availability of resources and personnel that might otherwise be occupied during on-duty hours may explain this phenomenon.

Thus far, studies focusing on traumatic OHCA are lacking. A study focusing on traumatic OHCA could yield different results, as traumatic OHCA not only has characteristics of both OHCA and trauma but has an extremely poor outcome. This information is important for identifying opportunities for quality improvement in emergency and critical care (e.g., staffing and design of the trauma and resuscitation care system) and to evaluate the effectiveness of Japan’s health policy.

To address this knowledge gap, we evaluated survival rates for traumatic OHCA by the time of day and day of the week. We hypothesized that there would be no differences in survival rates from traumatic OHCA regardless of the time of day (day/evening vs. night) and day of the week (weekday vs. weekend) in recent years, because of the Japanese government’s policy efforts to enhance the emergency care system (e.g., increasing the number of emergency and critical care centers).

## 2. Methods

### 2.1. Study Design and Data Source

This study was a registry-based analysis of patients with traumatic OHCA in Japan. The All-Japan Utstein Registry is a government-led nationwide population-based registry of OHCA patients, sponsored by the Fire and Disaster Management Agency (FDMA) of the Ministry of Internal Affairs and Communications. As was previously described [7,8,9], trained emergency medical service (EMS) personnel prospectively collected data on all OHCA patients who were transported to an emergency hospital using Utstein-style uniform reporting [19,20]. During the study period, almost all OHCA patients in Japan were included in this registry regardless of whether they had given “do not resuscitate” (DNR) orders or not, because EMS personnel were not allowed to terminate out-of-hospital resuscitation except in specific situations (e.g., decapitation, rigor mortis, livor mortis, or decomposition) in Japan.

The FDMA integrated data collected from 1-1-9 dispatch centers, fire stations, and receiving hospitals into the All-Japan Utstein Registry system on the FDMA database server. The logical internal checks with standardized software and certification of the FDMA secured the integrity, accuracy, and completeness of the data.

### 2.2. Study Setting and Population

Japan comprises an area of approximately 378,000 km^2^ and a population of approximately 126 million. Japan has a uniform nationwide EMS system with universal coverage [21,22]. Emergency number 1-1-9 is free for anyone who needs an ambulance and is available anytime and anywhere. Ambulance teams are provided by municipal governments through local fire departments, and there were approximately 730 fire departments with dispatch centers during the study period [6]. Physician-staffed ambulances are not usually available. Instead, most ambulances include at least one emergency life-saving technician (ELST) that is highly trained EMS personnel who can perform some part of advanced life support (ALS), although EMS personnel have different authorities depending on their completed training. EMS personnel in Japan perform cardiopulmonary resuscitation (CPR) following the Japanese CPR guidelines, which conform to the International Liaison Committee on Resuscitation (ILCOR) Consensus on Science with Treatment Recommendations (CoSTR). The EMS personnel perform prehospital ALS following a protocol fixed by each municipality (i.e., detailed protocols can vary among municipalities) based on the instructions of medical directors. However, even if they are especially trained ELSTs, EMS personnel are not allowed to perform surgical procedures (e.g., surgical airway, chest drain, pericardial drainage, or thoracotomy).

Critically ill and injured patients, including traumatic OHCA patients, are usually transported to a tertiary emergency medical center, called an emergency and critical care center, which covers 500,000 people in each region. Emergency and critical care centers are staffed with emergency and critical care physicians/surgeons, nurses, and other specialists and are operated 24 h a day, 7 days a week for critically ill and injured patients. These centers have a similar level of capacity for sufficient treatment. As of 2017, there were 286 adult emergency and critical care centers and 14 pediatric emergency and critical care centers in Japan (Table S1 in the Supplementary Materials) [14].

**Figure 1:**
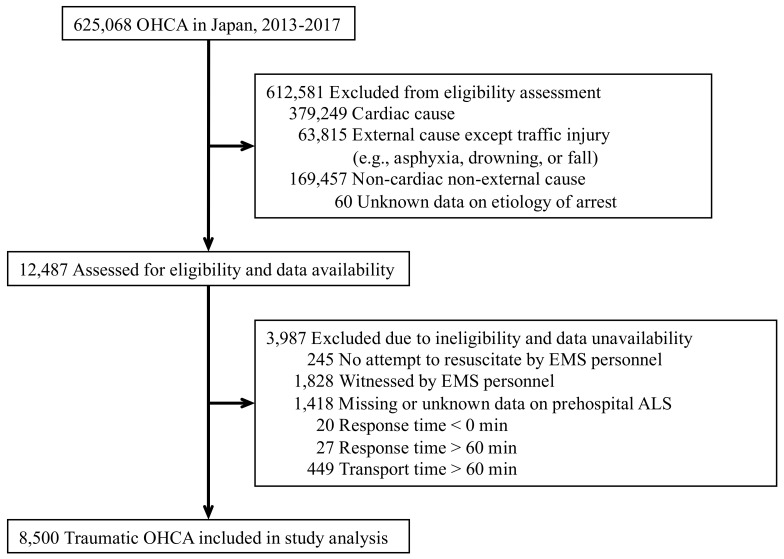
Patient flow diagram. Abbreviations: ALS, Advanced life support; EMS, Emergency medical service; OHCA, Out-of-hospital cardiac arrest.

This study included patients with traumatic OHCA following a traffic accident submitted to the All-Japan Utstein Registry between 1 January 2013, and 31 December 2017. We excluded patients for whom no CPR was attempted by EMS personnel or patients for whom cardiac arrest events were witnessed by EMS personnel. Additionally, we excluded patients with unrealistic or contradictory responses (i.e., response time < 0 min), and patients who did not receive timely treatments (i.e., response time > 60 min or transport time > 60 min). Patients with missing, incomplete, or inconsistent data, which accounted for less than 1% of all OHCA patients, were also excluded from the analyses (Figure 1).

This study was approved by the institutional review board of University of the Ryukyus, with a waiver of informed consent because of the anonymous nature of the data, and was conducted following the amended Declaration of Helsinki.

### 2.3. Variables

Data on patient characteristics (i.e., age and sex), bystander characteristics (i.e., witness, bystander CPR, public-access defibrillation, and dispatcher-assisted CPR), cardiac arrest characteristics (i.e., initial rhythm and etiology of arrest), event characteristics (i.e., time and place of arrest; for seasons and regions, the classification defined by the Japan Meteorological Agency was used), and prehospital ALS characteristics (i.e., intravenous line insertion, epinephrine administration, advanced airway management, and physician involvement in prehospital ALS) were collected. Data on a series of EMS activity times (i.e., emergency call, contact with patient, and hospital arrival) were recorded by each EMS squad. Subsequently, the response time was calculated as the time interval between emergency call and contact with patient, and transport time as the time interval between contact with patient and hospital arrival, based on time variables recorded in whole minutes. In our study, day/evening was defined as 7:00 a.m. to 22:59 p.m., and night was defined as 23:00 p.m. to 6:59 a.m. Weekdays were defined as Monday through Friday, and weekends were defined as Saturday and Sunday. To collect outcome data, a one-month follow-up survey was conducted by each fire department based on an inquiry for the receiving hospital. At that time, the etiology of cardiac arrest was also reconfirmed. If the patient was transferred or discharged from the hospital within one month after the event, further investigations were conducted by the fire department in cooperation with the hospital personnel.

### 2.4. Outcomes

The primary outcome of our analysis was survival one month after the event. The secondary outcome was prehospital return of spontaneous circulation (ROSC).

### 2.5. Statistical Analysis

Descriptive statistics were used to characterize traumatic OHCA patients according to time of day and day of the week. Categorical variables were presented as counts with proportions, and differences between groups were evaluated using the χ^2^ test. Continuous variables were presented as means with standard deviations (SDs) or medians with interquartile ranges (IQRs), and differences between the groups were evaluated using the *t*-test or the Wilcoxon Mann–Whitney test, respectively.

To examine the independent association of both time of day (day/evening vs. night) and day of the week (weekday vs. weekend) with outcomes after traumatic OHCA, multivariable logistic regression models were used. For multivariable logistic regression models, the following variables that could influence the outcomes were included: age, sex, witness (unwitnessed, witnessed by a family member, or witnessed by a non-family member), bystander CPR, public-access defibrillation, initial rhythm (ventricular fibrillation, ventricular tachycardia, pulseless electrical activity, asystole, or others), prehospital ALS (basic life support (BLS) only, ALS by EMS personnel, or ALS by physician), response time, transport time, year of arrest, season of arrest (spring, summer, autumn, or winter), region of arrest (north, east, west, or south), time of day (day/evening or night), and day of the week (weekday or weekend). Adjusted odds ratios (ORs) with 95% confidence intervals (CIs) were reported.

To further characterize the association between time of day (day/evening vs. night) and one-month survival after traumatic OHCA, we additionally conducted subgroup analyses according to several predefined subgroups: age (<18 years, or 18–64 years, or ≥65 years) and prehospital resuscitation (BLS only, ALS by EMS personnel, or ALS by physician). Multivariable logistic regression models included the same set of variables used in the primary analyses, and adjusted ORs of one-month survival for patients during the day/evening vs. night were reported with 95% CIs.

JMP Pro 15.0.0 software (SAS Institute Inc., Cary, NC, USA) was used for the statistical analyses. A two-sided *p* value of 0.05 was considered statistically significant for all hypothesis tests.

## 3. Results

During the study period, we identified 8500 eligible patients with traumatic OHCA (Figure 1). The baseline characteristics are shown in Table 1. 6233 events (73.3%) occurred during the day/evening hours, and 2267 events (26.7%) occurred during the night hours. 6018 events (70.8%) occurred on weekdays, and 2482 events (29.2%) occurred on weekends.

Patients experiencing OHCA during the night were significantly younger than patients during the day/evening, and most of them (79.9%) were male. Compared with during the day/evening, fewer patients had a witnessed event or received bystander CPR during the night. Furthermore, patients during the night had a lower chance of receiving ALS (especially, ALS by physician) in the prehospital setting than patients during the day/evening. In the comparison between weekdays and weekends, there were few, if any, differences between the two groups.

**Figure 2:**
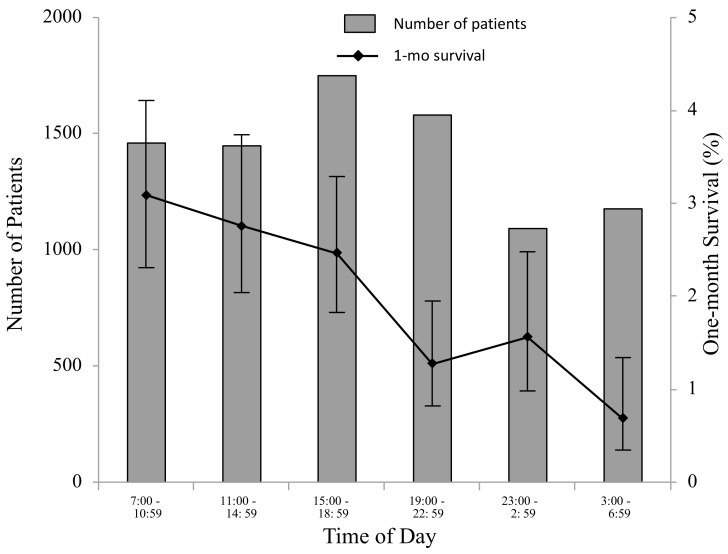
Number of patients and rate of one-month survival by time of day. Time of day was categorized into six periods according to 4-h intervals. One-month survival was highest during 7:00 a.m. to 10:59 a.m. and lowest during 3:00 a.m. to 6:59 a.m. (*p* < 0.0001).

**Figure 3:**
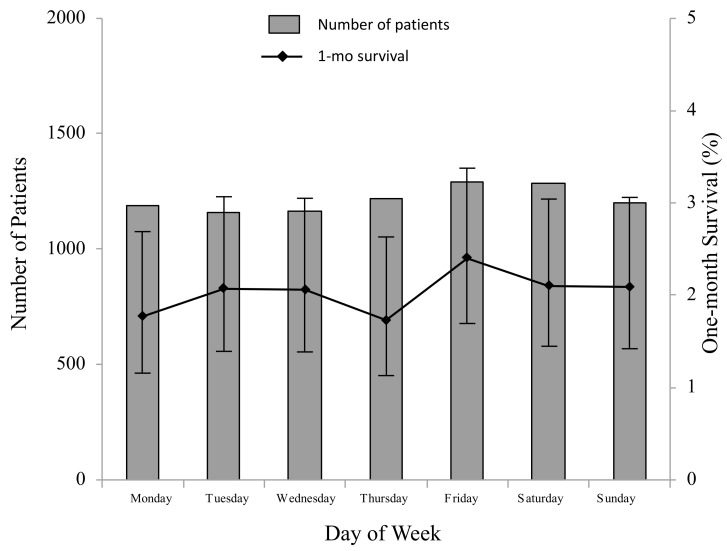
Number of patients and rate of one-month survival by day of week. No difference was observed in one-month survival between the days of the week (*p* = 0.9273).

Of the 8500 traumatic OHCA patients, 173 (2.0%) survived one month after OHCA. Unadjusted one-month survival was characterized by the time of day (categorized into six periods according to 4-hour intervals) in Figure 2 and by the day of the week in Figure 3. One-month survival was highest during 7:00 a.m. to 10:59 a.m. and lowest during 3:00 a.m. to 6:59 a.m. (*p* < 0.0001). No difference was observed in one-month survival between the days of the week (*p* = 0.9273).

Table 2 and Table 3 present the outcomes of traumatic OHCA patients according to time of day (day/evening vs. night) and day of the week (weekday vs. weekend), respectively. After adjusting for potential confounders, one-month survival during the day/evening (148/6233 [2.4%]) was significantly higher than during the night (25/2267 [1.1%]) (adjusted OR, 1.95 [95%CI, 1.24–3.07]), whereas there was no significant difference in one-month survival between weekdays (121/6018 [2.0%]) and weekends (527/2482 [2.1%]) (adjusted OR, 0.97 [95%CI, 0.69–1.38]). Similar associations were observed for prehospital ROSC.

**Figure 4:**
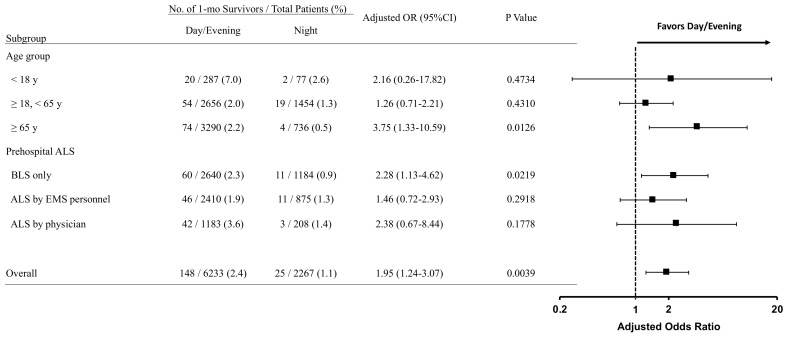
Adjusted odds ratios of one-month survival for the prespecified subgroups. The associations between time of day (day/evening vs night) and one-month survival after traumatic OHCA were reported as adjusted ORs with 95% CIs for the prespecified subgroups according to age (<18 years, 18–64 years, or ≥65 years) and prehospital ALS (no ALS [BLS only], ALS by EMS personnel, or ALS by physician). Multivariable logistic regression models were performed by including the same set of variables used in the primary analyses. Abbreviations: ALS, Advanced life support; BLS, Basic life support; CI, Confidence interval; EMS, Emergency medical service; OHCA, Out-of-hospital cardiac arrest; OR, Odds ratio.

In the subgroup analyses (Figure 4), time of day (day/evening vs. night) was significantly associated with one-month survival in the elderly (≥65 years) (2.2% vs. 0.5%; adjusted OR, 3.75 (95%CI, 1.33–10.59)) or patients who did not receive prehospital ALS (2.3% vs. 0.9%; adjusted OR, 2.28 (95%CI, 1.13–4.62)).

## 4. Discussion

In this nationwide population-based observational study of traumatic OHCA, the rate of one-month survival was lower at night compared with day/evening, whereas there was no difference in one-month survival between weekdays and weekends, after adjusting for potential confounders. Although the observational study design precludes ascertainment of causality, the large sample size based on a government-led nationwide population-based registry that routinely collected data for all OHCA patients transported to an emergency hospital ensures the statistical robustness of our findings.

To the best of our knowledge, this is the first study to examine the association between nights and weekends and one-month survival in traumatic OHCA. Several high-quality studies can be identified by expanding the scope of the search from traumatic OHCA to include non-traumatic OHCA and trauma without cardiac arrest [15,16,17,18]. Previous studies focusing on OHCA (including non-trauma cases) showed that the rate of survival was lower during nights than during days/evenings, and there was no difference between weekends and weekdays [15,16,17]. On the other hand, previous studies focusing on trauma (including non-cardiac arrest cases) showed that there was no difference in survival rate between nights and days/evenings, and the rate of survival was higher during weekends than weekdays [15,18]. Our findings are consistent with previous studies of OHCA, regardless of the difference in etiology of cardiac arrest, and inconsistent with the findings of previous trauma studies. This may imply that traumatic OHCA is strongly characterized by OHCA rather than trauma.

Several reasons were considered as to why the night was associated with a decreased chance of survival after traumatic OHCA, even when no difference was observed between weekdays and weekends. Although Japan’s health policy has promoted the establishment of more emergency and critical care centers that can provide advanced and highly specialized care 24 h a day, 365 days a year, it may have been more difficult to correct the disparities between night and day/evening than between weekends and weekdays. In general, hospital staff, especially the number of senior healthcare professionals, is reduced during the night, although the intensity or level of staffing seems to be associated with hospital mortality [23,24,25]. In addition, the night shift can affect healthcare professionals’ cognitive and psychomotor performance and may increase clinically significant medical errors [26,27]. During the night, the availability of resources and personnel may also be restricted. It is important to identify potential causes for the decreased survival after traumatic OHCA during the night to determine whether some measures can be taken against the causes. This warrants further investigation.

Even if increasing facilities and healthcare professionals for trauma resuscitation is effective in improving survival after traumatic OHCA, there is a limit to this plan because of limited human resources. Over the past few decades, the Japanese government has promoted medical care plans to increase emergency and critical care centers that can provide advanced and highly specialized care for critically ill and injured patients 24 h a day, 365 days a year to enhance the trauma care system (Table S1 in the Supplementary Materials). Then again, in recent years, the Japanese government has also promoted work style reform (including regulation of long working hours for physicians) [28,29,30,31]. In the face of such contradicting issues, there are high hopes that task shifting and task sharing will resolve the complex problem [32,33]. In this regard, our subgroup analysis demonstrated interesting findings (Figure 4). The survival disparities between night and day/evening were eliminated when prehospital ALS was performed, regardless of the type of healthcare professionals. Further research will be required to practice strategic staffing not only in the emergency department, operating room, and intensive care unit but also in the prehospital setting.

## 5. Limitations

Several limitations should be considered when interpreting our findings. First, the observational study design could only derive association rather than causality despite efforts to control for confounders, as the possibility of residual selection bias and unmeasured confounders remains. Second, the generalizability of our findings to other countries is unclear. The populations of patients with traumatic OHCA may vary by country. Moreover, different EMS systems or hospital staffing patterns during the night could yield different results. It is also unclear whether our findings can be generalized to any patients with traumatic OHCA. In our subgroup analyses, there were no survival disparities between night and day/evening among patients <65 years or patients who received prehospital ALS, although we acknowledge that the point estimates for the treatment effect favoring day/evening may have been statistically different with larger sample size. Third, the All-Japan Utstein Registry data lack information on in-hospital or post-resuscitation care. Although the data allow for adjustment for a large number of potential confounders, unmeasured confounders, including inpatient data, cannot be adjusted for. Fourth, the associations between off-duty hours and neurological outcomes after traumatic OHCA were unclear in our study. The sample was likely too small to detect differences in neurological outcomes (Tables S2 and S3 in the Supplementary Materials). Fifth, our study was not designed to identify the underlying causes for survival disparities between on-duty hours and off-duty hours, although our study highlights an important public health concern. Further studies are required to determine the mechanisms that cause survival disparities between night and day/evening, which may have important implications for hospital staffing, shift scheduling, training, or resource allocation. Finally, this study lacks an economic perspective. A formal cost-effectiveness study would be required before putting the plan into practice, even if further studies reveal that appropriate hospital staffing, resource allocation, or education can eliminate survival disparities between night and day/evening.

## 6. Conclusions

In conclusion, we found that the rate of one-month survival after traumatic OHCA was lower at night compared with day/evening, whereas there was no difference between weekends and weekdays, after adjusting for potential confounders. Although there are several plausible mechanisms that cause survival disparities between night and day/evening, the underlying causes of survival disparities should be explored through further research. A clearer understanding of the reasons for such disparities will play an important role in policy making to achieve parity in treatment outcomes between on-duty hours and off-duty hours among critically ill and injured patients.

## Figures and Tables

**Table 1:** Baseline characteristics according to time of day and day of week in the full cohort.

Characteristic	All Patients n = 8500	Time of Day			Day of Week		
		Day/Evening n = 6233	Night n = 2267	*p* Value	Weekday n = 6018	Weekend n = 2482	*p* Value
Baseline Characteristics							
Age, years							
– Mean (SD)	57.7 (22.3)	60.0 (22.3)	51.3 (21.0)	<0.0001	59.0 (22.1)	54.6 (22.5)	<0.0001
– Median (IQR)	63 (41–76)	66 (45–78)	52 (33–69)	<0.0001	65 (43–77)	58 (37–74)	<0.0001
(1) < 18 years—No. (%)	364 (4.3)	287 (4.6)	77 (3.4)	<0.0001	239 (4.0)	125 (5.0)	<0.0001
(2) ≥ 18, < 65 y—No. (%)	4110 (48.4)	2656 (42.6)	1454 (64.1)		2759 (45.8)	1351 (54.4)	
(3) ≥65 years—No. (%)	4026 (47.4)	3290 (52.8)	736 (32.5)		3020 (50.2)	1006 (40.5)	
Sex							
(1) Male—No. (%)	5828 (68.6)	4016 (64.4)	1812 (79.9)	<0.0001	4029 (66.9)	1799 (72.5)	<0.0001
(2) Female—No. (%)	2672 (31.4)	2217 (35.6)	455 (20.1)		1989 (33.1)	683 (27.5)	
Witness							
(1) No witness—No. (%)	2795 (32.9)	1943 (31.2)	852 (37.6)	<0.0001	1982 (32.9)	813 (32.7)	0.6047
(2) By family member—No. (%)	335 (3.9)	304 (4.9)	31 (1.4)		229 (3.8)	106 (4.3)	
(3) By non–family member—No. (%)	5370 (63.2)	3986 (63.9)	1384 (61.0)		3807 (63.3)	1563 (63.0)	
Bystander CPR							
(1) Yes–No. (%)	2096 (24.7)	1709 (27.4)	387 (17.1)	<0.0001	1431 (23.8)	665 (26.8)	0.0034
(2) No–No. (%)	6404 (75.3)	4524 (72.6)	1880 (82.9)		4587 (76.2)	1817 (73.2)	
Public–access defibrillation							
(1) Yes–No. (%)	25 (0.3)	20 (0.3)	5 (0.2)	0.4501	18 (0.3)	7 (0.3)	0.8949
(2) No–No. (%)	8475 (99.7)	6213 (99.7)	2262 (99.8)		6000 (99.7)	2475 (99.7)	
Dispatcher’s instruction for CPR							
(1) Yes–No. (%)	1703 (20.0)	1304 (20.9)	399 (17.6)	0.0007	1193 (19.8)	510 (20.5)	0.4483
(2) No–No. (%)	6797 (80.0)	4929 (79.1)	1868 (82.4)		4825 (80.2)	1972 (79.5)	
Initial rhythm							
(1) VF—No. (%)	151 (1.8)	113 (1.8)	38 (1.7)	<0.0001	107 (1.8)	44 (1.8)	0.8400
(2) VT—No. (%)	11 (0.1)	10 (0.1)	1 (0.0)		8 (0.1)	3 (0.1)	
(3) PEA—No. (%)	2954 (34.7)	2268 (36.4)	686 (30.3)		2113 (35.1)	841 (33.9)	
(4) Asystole—No. (%)	5191 (61.1)	3682 (59.1)	1509 (66.6)		3657 (60.8)	1534 (61.8)	
(5) Others (e.g., Bradycardia)—No. (%)	193 (2.3)	160 (2.6)	33 (1.4)		133 (2.2)	60 (2.4)	
Prehospital ALS							
(1) BLS only—No. (%)	3824 (45.0)	2640 (42.3)	1184 (52.2)	<0.0001	2675 (44.5)	1149 (46.3)	0.0760
(2) ALS by EMS personnel—No. (%)	3285 (38.6)	2410 (38.7)	875 (38.6)		2325 (38.6)	960 (38.7)	
(3) ALS by physician—No. (%)	1391 (16.4)	1183 (19.0)	208 (9.2)		1018 (16.9)	373 (15.0)	
Response time, min							
—Mean (SD)	10.5 (6.1)	10.6 (5.9)	10.5 (6.3)	0.4011	10.4 (5.9)	10.8 (6.4)	0.0034
—Median (IQR)	9 (7–12)	9 (7–12)	9 (7–12)	0.0754	9 (7–12)	9 (7–13)	0.0153
Transport time, min							
—Mean (SD)	26.1 (11.4)	26.4 (11.6)	25.3 (11.1)	<0.0001	26.1 (11.5)	26.0 (11.4)	0.5774
—Median (IQR)	24 (18–33)	24 (18–33)	23 (17–31)	<0.0001	24 (17–33)	24 (18–33)	0.6613
Year of arrest							
(1) 2013—No. (%)	1751 (20.6)	1266 (20.3)	485 (21.4)	0.1679	1257 (20.9)	494 (19.9)	0.5670
(2) 2014—No. (%)	1696 (19.9)	1216 (19.5)	480 (21.2)		1178 (19.6)	518 (20.9)	
(3) 2015—No. (%)	1700 (20.0)	1254 (20.1)	446 (19.7)		1208 (20.1)	492 (19.8)	
(4) 2016—No. (%)	1690 (19.9)	1270 (20.4)	420 (18.5)		1187 (19.7)	503 (20.3)	
(5) 2017—No. (%)	1663 (19.6)	1227 (19.7)	436 (19.2)		1188 (19.7)	475 (19.1)	
Season of arrest							
(1) Spring (March, April, May)—No. (%)	1997 (23.5)	1466 (23.5)	531 (23.4)	0.9200	1426 (23.7)	571 (23.0)	0.5902
(2) Summer (June, July, August)—No. (%)	1906 (22.4)	1404 (22.5)	502 (22.2)		1333 (22.2)	573 (23.1)	
(3) Autumn (September, October, November)—No. (%)	2229 (26.2)	1639 (26.3)	590 (26.0)		1566 (26.0)	663 (26.7)	
(4) Winter (December, January, February)—No. (%)	2368 (27.9)	1724 (27.7)	644 (28.4)		1693 (28.1)	675 (27.2)	
Region of arrest							
(1) North—No. (%)	906 (10.7)	729 (11.7)	177 (7.8)	<0.0001	646 (9.9)	260 (10.5)	0.5502
(2) East—No. (%)	4588 (54.0)	3281 (52.6)	1307 (57.7)		3269 (54.2)	1319 (53.1)	
(3) West—No. (%)	2910 (34.2)	2175 (34.9)	735 (32.4)		2039 (34.1)	871 (35.1)	
(4) South—No. (%)	96 (1.1)	48 (0.8)	48 (2.1)		64 (1.8)	32 (1.3)	

The data are expressed as the number (%) of patients, mean (SD), or median (IQR), unless otherwise indicated. Abbreviations: ALS, Advanced life support; BLS, Basic life support; CPR, Cardiopulmonary resuscitation; EMS, Emergency medical service; IQR, Interquartile range; PEA, pulseless electrical activity; SD, Standard deviation; VF, Ventricular fibrillation; VT, Ventricular tachycardia.

**Table 2:** Outcomes for traumatic OHCA during the day/evening vs. night.

Outcome	Day/Evening n = 6233	Night n = 2267	Adjusted OR (95%CI)	*p* Value
One–month survival—No. (%)	148 (2.4)	25 (1.1)	1.95 (1.24–3.07)	0.0039
Prehospital ROSC—No. (%)	407 (6.5)	72 (3.2)	1.56 (1.18–2.06)	0.0017

The data are expressed as the number (%) of patients, unless otherwise indicated. The associations between time of day (day/evening vs. night) and outcomes after traumatic OHCA were reported as adjusted ORs with 95% CIs. Abbreviations: CI, Confidence interval; OHCA, Out-of-hospital cardiac arrest; OR, Odds ratio; ROSC, Return of spontaneous circulation.

**Table 3:** Outcomes for traumatic OHCA during weekday vs. weekend.

Outcome	Weekday n = 6018	Weekend n = 2482	Adjusted OR (95% CI)	*p* Value
One-month survival—No. (%)	121 (2.0)	52 (2.1)	0.97 (0.69–1.38)	0.8712
Prehospital ROSC—No. (%)	354 (5.9)	125 (5.0)	1.19 (0.95–1.49)	0.1351

The data are expressed as the number (%) of patients, unless otherwise indicated. The associations between day of week (weekday vs. weekend) and outcomes after traumatic OHCA were reported as adjusted ORs with 95% CIs. Abbreviations: CI, Confidence interval; OHCA, Out-of-hospital cardiac arrest; OR, Odds ratio; ROSC, Return of spontaneous circulation.

## Data Availability

The All-Japan Utstein Registry data were supplied by the Fire and Disaster Management Agency of the Ministry of Internal Affairs and Communications under license, and so cannot be made freely available. Requests for access to these data should be made to the Fire and Disaster Management Agency.

## Supplementary Materials

The following are available online at https://www.mdpi.com/article/10.3390/ijerph182312769/s1, Table S1: Change in number of emergency and critical care centers in Japan, 2005–2017, Table S2: Neurologically favorable survival for traumatic OHCA during the day/evening vs. night, Table S3: Neurologically favorable survival for traumatic OHCA during weekday vs. weekend.

## Abbreviations

ALS: Advanced life support; BLS: Basic life support; CoSTR: Consensus on Science with Treatment Recommendations; CPR: Cardiopulmonary resuscitation; DNR: Do not resuscitate; ELST: Emergency life-saving technician; EMS: Emergency medical service; FDMA: Fire and Disaster Management Agency; ILCOR: International Liaison Committee on Resuscitation; OHCA: Out-of-hospital cardiac arrest; ROSC: Return of spontaneous circulation.
