# Supplementary material for: Association of Nights and Weekends with Survival of Traumatic Out-of-Hospital Cardiac Arrest following Traffic Collisions: Japanese Registry-Based Study
Source: Int J Environ Res Public Health. 2021 Dec 3;18(23):12769. doi: 10.3390/ijerph182312769 (PMC8657123; doi:10.3390/ijerph182312769)
Supplement: Supplementary file 1 [file ijerph-18-12769-s001.zip › ijerph-1445753-supplementary.pdf]

Supplementary Appendix

This appendix has been provided by the authors to give readers additional information about their work.

Table of Contents

Table S1. Change in number of emergency and critical care centers in Japan, 2005-2017……………………………………………………………………………………2
Table S2. Neurologically favorable survival for traumatic OHCA during day/evening vs night……………………………………………………………………………………3
Table S3. Neurologically favorable survival for traumatic OHCA during weekday vs weekend……………………………………………………………………………………4

Table S1. Change in number of emergency and critical care centers in Japan, 2005-2017

	2005	2006	2007	2008	2009	2010	2011	2012	2013	2014	2015	2016	2017
Emergency and Critical Care Center	178	189	201	208	214	221	235	249	259	266	271	279	286
Pediatric Emergency and Critical Care Center	1	1	2	2	2	4	4	4	6	8	8	11	14

In Japan, an emergency and critical care center, which is staffed with a trauma team, emergency and critical care physicians/surgeons, nurses, and other specialists and is operated 24 hours a day, 7 days a week for critically ill and injured patients, serves as a level 1 trauma center. A pediatric emergency and critical care center is specialized in pediatric care. Over the past few decades, the Japanese government has promoted medical care plans to increase the number of adult and pediatric emergency and critical care centers.

Table S2. Neurologically favorable survival for traumatic OHCA during day/evening vs night

Outcome	Day/Evening n = 6,233	Night n = 2,267	Adjusted OR (95%CI)	*P* value
Favorable Neurological outcome (CPC 1 or 2) - No. (%)*	36 (0.6)	11 (0.5)	0.76 (0.35-1.64)	0.4864
1) CPC 1 - No. (%)	21 (0.3)	9 (0.4)	NA	NA
2) CPC 2 - No. (%)	15 (0.2)	2 (0.1)	NA	NA
3) CPC 3 - No. (%)	40 (0.7)	2 (0.1)	NA	NA
4) CPC 4 - No. (%)	61 (1.0)	10 (0.4)	NA	NA
5) CPC 5 - No. (%)	6085 (97.6)	2242 (98.9)	NA	NA
6) CPC Unknown - No. (%)	11 (0.2)	2 (0.1)	NA	NA

The data are expressed as the number (%) of patients, unless otherwise indicated.

The association between time of day (day/evening vs night) and one-month neurologically favorable survival after traumatic OHCA was reported as adjusted OR with 95% CI.

*13 patients for whom one-month neurological status was not available were excluded from analysis

Abbreviations: CI, Confidence interval; CPC, Cerebral performance category; OHCA, Out-of-hospital cardiac arrest; OR, Odds ratio

Table S3. Neurologically favorable survival for traumatic OHCA during weekday vs weekend

Outcome	Weekday n = 6,018	Weekend n = 2,482	Adjusted OR (95%CI)	*P* value
Favorable Neurological outcome (CPC 1 or 2) - No. (%)*	31 (0.5)	16 (0.6)	0.85 (0.44-1.66)	0.6348
1) CPC 1 - No. (%)	21 (0.3)	9 (0.4)	NA	NA
2) CPC 2 - No. (%)	10 (0.2)	7 (0.3)	NA	NA
3) CPC 3 - No. (%)	29 (0.5)	13 (0.5)	NA	NA
4) CPC 4 - No. (%)	49 (0.8)	22 (0.9)	NA	NA
5) CPC 5 - No. (%)	589 (98.0)	243 (97.9)	NA	NA
6) CPC Unknown - No. (%)	12 (0.2)	1 (0.0)	NA	NA

The data are expressed as the number (%) of patients, unless otherwise indicated.

The association between day of week (weekday vs weekend) and one-month neurologically favorable survival after traumatic OHCA was reported as adjusted OR with 95% CI.

*13 patients for whom one-month neurological status was not available were excluded from analysis

Abbreviations: CI, Confidence interval; CPC, Cerebral performance category; OHCA, Out-of-hospital cardiac arrest; OR, Odds ratio
